# Protocol for Comparing the Efficacy of Three Reconstruction Methods of the Digestive Tract (Kamikawa Versus Double-Tract Reconstruction Versus Tube-Like Stomach) After Proximal Gastrectomy

**DOI:** 10.3389/fsurg.2022.891693

**Published:** 2022-05-25

**Authors:** Wei Dong Wang, Rui Qi Gao, Tong Chen, Dan Hong Dong, Qin Chuan Yang, Hai Kun Zhou, Jiang Peng Wei, Peng Fei Yu, Xi Sheng Yang, Xiao Hua Li, Gang Ji

**Affiliations:** Department of Gastrointestinal Surgery, Xijing Hospital, Air Force Military Medical University, Xi’an, China

**Keywords:** gastric cancer, Kamikawa, double-tract reconstruction, tube-like stomach, proximal gastrectomy, protocol

## Abstract

**Background:**

Appropriate gastrointestinal reconstruction after proximal gastrectomy can effectively reduce the incidence of postoperative complications in patients with proximal early gastric cancer. However, there is still great controversy about the choice of digestive tract reconstruction after proximal gastrectomy, and there is no clinical consensus on the choice of digestive tract reconstruction after proximal gastrectomy. Currently, there is a lack of large-sample, prospective, randomized controlled studies to compare the efficacy of Kamikawa, double-tract reconstruction, and tube-like stomach reconstruction after proximal gastrectomy.

**Methods/design:**

This study will investigate the efficacy of three reconstruction methods after proximal gastrectomy in a prospective, multicenter, randomized controlled trial, which will enroll 180 patients with proximal early gastric cancer. Patients will be randomly divided into three groups: Group A (Kamikawa, *n* = 60), Group B (double-tract reconstruction, *n* = 60), and Group C (tube-like stomach, *n* = 60). The general information, past medical history, laboratory findings, imaging findings, and surgical procedures of the patients will be recorded and analyzed. The incidence of reflux esophagitis will be recorded as the primary endpoint. The incidence of anastomotic leakage, anastomotic stenosis, operative time and intraoperative blood loss will be recorded as secondary endpoints.

**Discussion:**

This study will establish a large-sample, prospective, randomized controlled trial to compare the efficacy of Kamikawa, double-tract reconstruction, and tube-like stomach reconstruction after proximal gastrectomy.

**Trial registration:**

This study was approved by the Chinese Clinical Trial Registry and registered on April 30, 2021. The registration number is ChiCTR2100045975.

## Background

Gastric cancer (GC) is one of the most common malignant tumors of the digestive system and the fifth most common cancer in the world, which seriously threatens human survival and health (1). Proximal early gastric cancer (EGC) refers to the cancers that occurs in the upper 1/3 of the stomach, including esophageal and gastric junction cancers (2). In recent years, the incidence of proximal EGC has been increasing (3).

At present, the treatment of proximal EGC is mainly through surgical means (4). Proximal gastrectomy, as a surgical method to preserve the gastric function of patients, has been widely used in clinical practice. Takiguchi et al. (5) showed that compared with total gastrectomy, proximal gastrectomy had more advantages in terms of weight loss, the necessity of additional meals, etc. However, proximal gastrectomy is associated with adverse outcomes, such as reflux esophagitis, dumping syndrome, and anorexia, which seriously affect the quality of life of patients after gastrectomy (6).

Appropriate reconstruction methods after proximal gastrectomy can effectively reduce the incidence of postoperative complications in patients with gastric cancer. Japanese guidelines recommend that commonly used methods of gastrointestinal reconstruction after proximal gastrectomy include esophagogastrotomy (EG), jejunal interposition (JI), jejunal pouch interposition (JPI) and double-tract reconstruction (DTR) (7). Kamikawa et al. (8) reported the first use of a double-muscle flap anastomosis after proximal gastrectomy in 2001, also known as Kamikawa anastomosis, could reduce reflux (9). Yasuda et al. (10) reported that tubular gastric reconstruction after proximal gastrectomy could reduce the incidence of reflux symptoms.

Recently, DTR has been widely used in gastrointestinal reconstruction after proximal gastrectomy due to its advantages in reducing the incidence of anastomotic fistula and reflux symptoms after proximal gastrectomy (11–13). However, the choice of the best method of digestive tract reconstruction after proximal gastrectomy is still controversial, and there is still no clinical consensus on the method of digestive tract reconstruction. Therefore, we urgently need a simple, safe digestive tract reconstruction method with better absorption and digestion function, to improve patients’ postoperative quality of life. Currently, in the specialized field of gastrointestinal reconstruction after proximal gastrectomy, there is a lack of large-sample, prospective, randomized controlled studies to compare the efficacy of Kamikawa, DTR, and tubular stomach reconstruction after proximal gastrectomy. To identify a standardized digestive tract reconstruction method after proximal gastrectomy for clinical applications, our center will conduct a large sample, prospective, multicenter RCT, comparing the efficacy of three reconstruction methods after proximal gastrectomy: Kamikawa, DTR and tube-like stomach.

## Methods/Design

**Figure 1:**
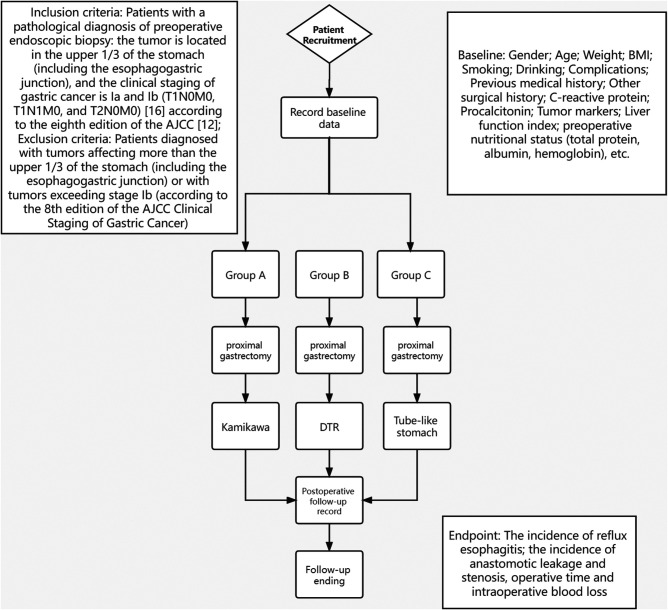
This is the whole flow diagram of the test.

This study will be a multicenter RCT in which 180 patients enrolled from May 2021 to November 2024, will be randomly assigned to Group A, Group B or Group C in a 1:1:1 allocation ratio. Figure 1 shows the study flow chart.

### Main Objective

A total of 180 patients will be grouped in a 1:1:1 allocation ratio to explore a comparison of the incidence of reflux esophagitis after three reconstruction surgeries (Kamikawa, DTR and tube-like stomach) applied after proximal gastrectomy according to the study process shown in Figure 1.

### Secondary Objectives

- To explore a comparison of the incidence of anastomotic leakage after three reconstruction surgeries (Kamikawa, DTR and tube-like stomach) applied after proximal gastrectomy
- To explore a comparison of the incidence of anastomotic stenosis after three reconstruction surgeries (Kamikawa, DTR and tube-like stomach) applied after proximal gastrectomy
- To explore a comparison of the operative time of three reconstruction surgeries (Kamikawa, DTR and tube-like stomach) applied after proximal gastrectomy
- To explore a comparison of the intraoperative blood loss of three reconstruction surgeries (Kamikawa, DTR and tube-like stomach) applied after proximal gastrectomy

### Patient Recruitment and Characteristics

The recruiting method is to collect patients screened by the investigators against the inclusion/exclusion criteria during a routine procedure. The researchers will direct them to sign informed consent forms. The above procedures follow the provisions of the Measures for Ethical Review of Biomedical Research Involving Human Beings (Trial), the Declaration of Helsinki v.08 and the International Ethical Guidelines for Biomedical Research Involving Human Beings.

### Inclusion Criteria

Patients who meet all of the following criteria will be enrolled:

- Patients aged 20–75 years, male or female;
- Patients with a pathological diagnosis of preoperative endoscopic biopsy: the tumor is located in the upper 1/3 of the stomach (including the esophagogastric junction), and the clinical staging of gastric cancer is Ia and Ib (T1N0M0, T1N1M0, and T2N0M0) (14) according to the eighth edition of the AJCC (15);
- Patients with no distant metastasis observed on preoperative chest radiograph, abdominal ultrasound or upper abdominal CT;
- Patients with ASA grade 1–3;
- Patients without contraindications to surgery;
- Patients and their families voluntarily signing the informed consent form and participating in the study;

### Exclusion Criteria

Patients will be excluded when meeting any of the following criteria:

- Patients diagnosed with primary tumors other than gastric adenocarcinoma;
- Patients diagnosed with tumors affecting more than the upper 1/3 of the stomach (including the esophagogastric junction) or with tumors exceeding stage Ib (according to the 8th edition of the AJCC Clinical Staging of Gastric Cancer) (15);
- Patients in which the tumor is located in the greater curvature side;
- Patients with distant metastasis;
- Patients with ASA ≥grade 4;
- Patients who have coagulation dysfunction and could not be corrected;
- Patients who were diagnosed with viral hepatitis and cirrhosis;
- Patients with diabetes mellitus, uncontrolled or controlled with insulin;
- Patients with organ failure such as heart, liver, lung, brain, kidney failure;
- Patients with ascites and cachexia preoperatively in poor general conditions;
- Patients refusing to sign the informed consent to participate in this study;
- Patients with immunodeficiency, immunosuppression or autoimmune diseases (such as allogeneic bone marrow transplant patients, immunosuppressive drugs, SLE, etc.).

### Terminating Study Criteria

The criteria for terminating the study are as follows:

- Patients who are unable to receive surgery due to all of these reasons once enrolled, and the reasons should be recorded;
- The investigators consider that the patients are not suitable to continue the clinical trial, and the reason needs to be recorded;
- Patients with unbearable adverse reactions or serious complications;
- Patients requesting termination of the trial;
- Patients violating the principles of the treatment.

### Participating Entities

As a multicenter study, the institutions included in this study were as follows: the First Affiliated Hospital of Air Force Military Medical University, the Second Affiliated Hospital of Air Force Medical University, the First Affiliated Hospital of Xi’an Jiaotong University, General Hospital of Ningxia Medical University and Henan Provincial People’s Hospital. These centers have rich experience in the clinical diagnosis and treatment of gastric cancer.

### Randomization Procedure

Eligible participants will be randomly assigned to Group A, Group B or Group C in a 1:1:1 allocation ratio. Randomized sequences will be generated by a biostatistician who will not be involved in this study using SAS software 9.2 (SAS Institute, Cary, NC, USA). The randomization list will be sealed in sequentially numbered opaque envelopes stored in a double-locked cabinet. Randomization will be performed by a research assistant who is not involved in recruitment. The envelopes will be stored separately after random allocation. Only data collection and analysis will be blinded because the participants and clinicians cannot be blinded to the intervention (16). Figure 1 shows the study flow chart.

### Treatment Protocols/Surgical Intervention

All eligible participants who undergo proximal gastrectomy will be randomly assigned to Group A, Group B or Group C in a 1:1:1 allocation ratio. Group A will receive reconstruction surgery with Kamikawa. Group B will receive reconstruction surgery with DTR. Group C will receive reconstruction surgery with a tube-like stomach. Table 1 shows the surgical methods applied in this study.

**Table 1:** This is the surgical methods applied in this study.

	proximal gastrectomy	Kamikawa	double-tract reconstruction	tube-like stomach
Group A	√	√	—	—
Group B	√	—	√	—
Group C	√	—	—	√

According to the Japanese gastric cancer treatment guidelines (7), routine abdominal exploration will be performed to confirm that no cancer cells have been detached, implanted or metastasized. The patients will undergo laparoscopic D2 lymphadenectomy in radical proximal gastrectomy. Based on the Chinese consensus on digestive tract reconstruction after proximal gastrectomy (17), different methods of digestive tract reconstruction will be performed for each group of patients. The three methods of digestive tract reconstruction are described as follows.

**Figure 2:**
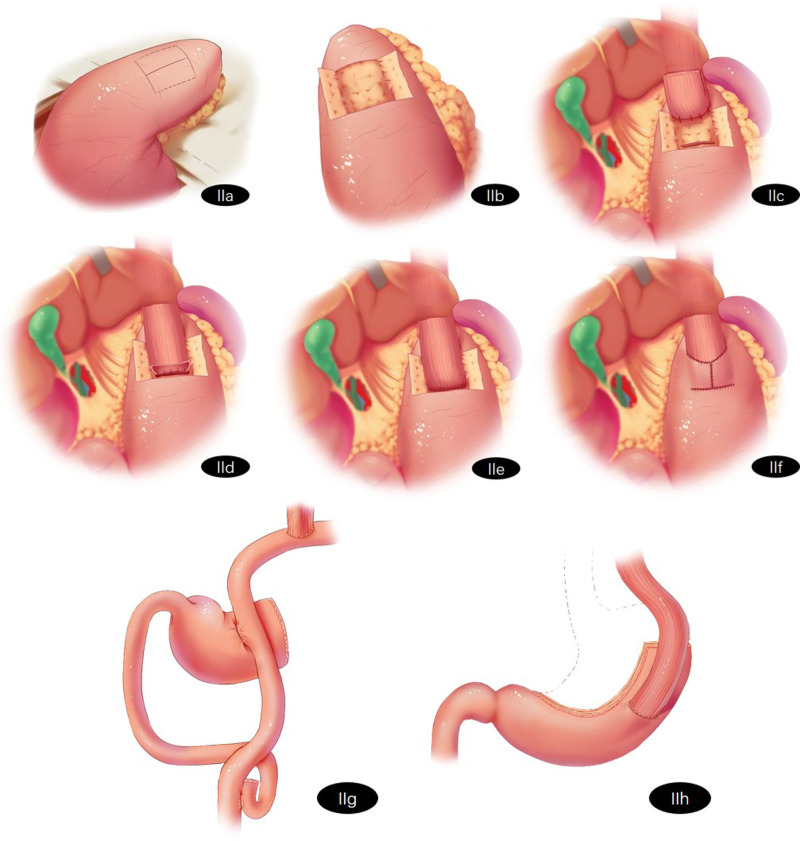
Schematic representation of reconstruction surgery of the digestive tract after proximal gastrectomy (**A**) Kamikawa: A “H"-shaped seromuscular flap (3.0 cm × 3.5 cm) will be marked on the anterior wall of the remnant stomach near the greater curvature of the stomach. (**B**) Kamikawa: The sarcoplasmic flap will be dissected between the submucosa and musculature. (**C**) Kamikawa: The gastric mucosa “window” with similar width to the esophagus will be made at the lower edge of the sarcoplasmic flap for anastomosis. Traction will be performed on the esophagus. Three to four stitches will be used to fix the posterior wall of the esophagus which is 5 cm away from the esophageal stump and the gastric stump at the upper edge of the sarcoplasmic flap. (**D**) Kamikawa: The whole esophagus will be sutured continuously with the gastric mucosa and submucosa. (**E**) Kamikawa: The whole anterior wall of the esophagus and the whole stomach will be sutured intermittently. (**F**) Kamikawa: Both sides of the sarcoplasmic flap will be sutured intermittently in the shape of a “Y” and fixed with the esophagus to cover the anastomosis. (**G**) Double-flap reconstruction (**H**) Tube-like stomach reconstruction.

The patients in Group A will receive Kamikawa. Lymph nodes will be routinely dissected. The esophagus will be transected, and the lower part of the esophagus will be dissociated at approximately 5 cm. The stomach will be dragged out through a 5 cm long incision made under the belly button or the xiphid process. The proximal stomach will be severed with a linear stapler 3 cm from the distal tumor. An “H”-shaped seromuscular flap (3.0 cm × 3.5 cm) will be marked on the anterior wall of the remnant stomach near the greater curvature of the stomach. The sarcoplasmic flap will be dissected between the submucosa and musculature, and the gastric mucosa “window” with a similar width as the esophagus will be made at the lower edge of the sarcoplasmic flap for anastomosis. Traction will be performed on the esophagus. Three to four stitches will be used to fix the posterior wall of the esophagus, which is 5 cm away from the esophageal stump and the gastric stump at the upper edge of the sarcoplasmic flap. The whole esophagus will be sutured continuously to the gastric mucosa and submucosa. The whole anterior wall of the esophagus and the whole stomach will be sutured intermittently. Both sides of the sarcoplasmic flap will be sutured intermittently in the shape of a “Y” and fixed with the esophagus to cover the anastomosis. The reconstruction process of Kamikawa is shown in Figures 2A–F.

The patients in Group B will receive double-flap reconstruction. Lymph nodes will be routinely dissected and the esophagus will be transected. The tumor and the proximal stomach will be removed. The jejunum and mesenteric vessels will be cut off 20–25 cm away from the Treitz ligament. The esophagus and distal jejunum will be anastomosed. A linear stapler will be used to close the broken jejunum, and the length of the blind-ending will remain 2–3 cm. The proximal jejunum and distal jejunum will be anastomosed at a distance of 45–50 cm beneath the esophagojejunostomy. Side-to-side anastomosis will be performed on the jejunum and the anterior wall of the remnant stomach 10–15 cm away from the anastomosis of the esophagojejunostomy. The resected end of the stomach will be closed. The reconstruction process of DTR is shown in Figure 2G.

The patients in Group C will receive tube-like stomach reconstruction. Lymph nodes will be routinely resected and the esophagus will be transected. The stomach will be cut off by a linear stapler. A curve will be made parallel to the greater curvature of the stomach (3–4 cm from the greater curvature of the stomach) along the side of the lesser curvature of the stomach from the gastric angle to the junction of the gastric fundus and gastric body. The cardia, tumor and part of the lesser curvature of the stomach will be removed. The length of the tube-like stomach is generally approximately 20 cm. The tissue of the lesser curvature of the stomach will be used to insert the stapler at the gastric corner taking care to not cut it off temporarily. The linear stapler will be inserted from the reserved small incision and pierced out from the anterior wall approximately 3.0 cm from the top of the remnant stomach. End-to-side esophagogastric anastomosis will be performed, and the small incision of the remnant stomach will be closed. The reconstruction process of the tube-like stomach is shown in Figure 2H.

### Clinical Data

The medical staff will acquire the clinical data of the patients, and record that on the online electronic platform (Http://www.medresman.org.cn) and in the CRF table. Only the attending physician will know the identity of the patients. The clinical data will include the patients’ general information, previous medical history, previous surgical history, laboratory examination findings, imaging findings, surgical procedures, the incidence of postoperative reflux esophagitis, the incidence of anastomotic leakage, the incidence of anastomotic stenosis, the incidence of postoperative complications, operative time, and intraoperative blood loss. The above results and information will be recorded in the CRF table. Preoperative and postoperative routine blood tests, biochemical indicators and tumor markers will be included in the laboratory examinations. The CRF table reflects a detailed description of the above data.

### Collection, Preservation and Management of the Biochemical Specimens

In this study, blood samples will be collected from the subjects and subsequently tested by the laboratory. All blood samples will be destroyed after testing and will not be stored.

### Sample Size Estimate and Statistical Analysis

Due to the lack of large sample studies comparing the efficacy of Kamikawa, DTR and tube-like stomach reconstruction methods after proximal gastrectomy, we could only estimate the sample size based on the data related to the incidence of postoperative reflux esophagitis in previous literatures. According to the research results of Kuroda et al. (9), the incidence of reflux esophagitis was 0 in patients who received Kamikawa reconstruction. Aburatani et al. (18) showed that the incidence of reflux esophagitis was 10.5% in patients who received DTR reconstruction. Yasuda et al. (10) demonstrated that the incidence of reflux esophagitis was 13.64% in patients who received a tube-like stomach. We designed a superiority study with a superiority margin of 5% (α = 0.05, β = 0.20, 80% power). Eligible participants will be randomly assigned to Group A, Group B or Group C in a 1:1:1 allocation ratio, and thus 53 patients per group will be required. Therefore, the total sample size is 159 for this study. Considering the loss to follow-up for various reasons, we selected the sample size to be 180.

### Study Endpoints

The primary endpoint will be the incidence of postoperative reflux esophagitis.

The secondary endpoints will be the incidence of anastomotic leakage, the incidence of anastomotic stenosis, the incidence of postoperative complications, operative time, and intraoperative blood loss of three reconstruction surgeries (Kamikawa, DTR and tube-like stomach) after proximal gastrectomy. The test and data collection progress of this experiment is shown in Table 2.

**Table 2:** This is the content of each follow-up which are sorted and merged.

Stage	Preoperation	Intraoperation	Postoperation!					
Follow up period	14-1days		1-14 days	1st month	3rd month	6th month	12th month	24th month
Baseline data collected	√	—	—	—	—	—	—	—
Inclusion and exclusion	√	—	—	—	—	—	—	—
Sign informed consent	√	—	—	—	—	—	—	—
Group determination	√	—	—	—	—	—	—	—
Fill in the basic information	√	—	—	—	—	—	—	—
Physical examination	√	—	—	—	—	—	—	—
Imaging examination	√	—	√	—	√	—	√	√
Laboratory examination	√	—	√	√	√	√	√	√
Operation information	—	√	—	—	—	—	—	—
Postoperative pathology	—	√	—	—	—	—	—	—
Safety observation	√	—	√	√	√	√	√	√
Operational observation	√	—	√	—	—	—	—	—
Record adverse events	√	—	√	√	√	√	√	√
Other works	√	√	√	√	√	√	√	√

### Follow-Up

The first follow-up visit will be arranged on the 14th postoperative day. Follow-up will include physical examination, laboratory examination, and imaging examination to determine the occurrence of postoperative complications. Patients will be followed up at 1, 3, 6, 12 and 24 months. The content of each follow-up will be sorted and merged, as presented in Table 2.

### Patient Protection/Written Informed Consent Forms

We will protect the patients’ personal information. We will never publicly disclose the patients’ information except as required by law. We will implement the informed consent process in strict accordance with relevant Chinese laws and regulations. We will obtain preapproval of the informed consent process for this study from the Internal Review Board/independent Ethics Committee prior to patient enrollment, including any changes made during the study. Informed consent will be obtained in writing from each patient once they are enrolled in the study before undergoing treatment. The original informed consent will be kept by the researchers, and additional copies will be given to the patients for the record.

### Monitoring of the Study

Prior to the start of the study, the project leader will visit each research center to discuss the project with its responsible personnel.

During the study, the project leader will regularly contact the project researchers to provide them with technical support and confirm whether the researchers are carrying out the study according to the plan. The project leader will verify the accuracy of the CRF data records by directly accessing the original records of each patient.

Representatives authorized by the project undertaker and the independent ethics committee may visit all centers for systematic and independent review to verify that all research-related practices are managed and that their data are recorded and accurately reported in accordance with the program GCP and ICH guidelines.

### Patient and Public Involvement

Patients and the public will not be involved in our studies for reporting, designing or implementing.

## Discussion

At present, proximal gastrectomy is increasingly used in the treatment of early proximal gastric cancer, and in some studies, it has shown a better therapeutic effect than total gastrectomy (5, 19–21). However, proximal gastrectomy could cause postoperative complications, especially reflux esophagitis, which has a serious impact on postoperative quality of life among patients (6, 22). Appropriate reconstruction of the digestive tract after proximal gastrectomy can effectively reduce the occurrence of these postoperative complications. Therefore, it is important to select the appropriate method of digestive tract reconstruction after proximal gastrectomy.

Currently, the commonly used methods of digestive tract reconstruction after proximal gastrectomy include EG, JI, JPI and DTR (6). However, there is still no standardized clinical consensus on selecting of reconstruction methods after proximal gastrectomy. Muraoka et al. (23) showed that Kamikawa reconstruction reduced the risk of reflux esophagitis after proximal gastrectomy by increasing the pressure on the lower esophagus of the patients. In their study, none of the patients undergoing Kamikawa reconstruction developed reflux symptoms, which proved that this method of reconstruction was safe and feasible. Aburatani et al. (18) showed that DTR, as one of the reconstruction methods after proximal gastrectomy, had an excellent effect in reducing the incidence of postoperative complications, especially in preventing reflux esophagitis and anastomotic stenosis. Shiraishi et al. (24) showed that a tube-like stomach could prevent reflux esophagitis after proximal gastrectomy by maintaining the basic anatomical structure of the stomach, and could improve the postoperative quality of life of patients compared with traditional esophagogastric anastomosis.

Although the methods above have their own individual advantages, there is still a lack of clinical research on Kamikawa, DTR and tube-like stomach reconstruction after proximal gastrectomy. This study will establish a large-sample, prospective, randomized controlled trial to compare the efficacy of Kamikawa, DTR, and tube-like stomach reconstruction after proximal gastrectomy. Our work may provide more evidence-based medical evidence for the selection of digestive tract reconstruction after proximal gastrectomy.

If the results of this research are in line with our expectations, it will provide a landmark reference for medical staff to improve the prognosis of patients with gastrointestinal reconstruction after proximal gastrectomy. We hope to gain experience from this study and integrate these results into clinical activities and create standardized treatment protocols to guide clinical practice and further improve the postoperative quality of life of these patients.

## Advantages and Limitations of This Study

Advantages: This is the first prospective large sample multicenter RCT to systematically compare the efficacy of three reconstruction methods of the digestive tract (Kamikawa versus DTR versus tube-like stomach) after proximal gastrectomy. Previous studies were mostly retrospective studies (25–27). A few prospective studies focused only on comparing the two reconstruction methods (28, 29).

Limitations: Japanese guidelines recommend that commonly used methods of gastrointestinal reconstruction after proximal gastrectomy include esophagogastrotomy (EG), jejunal interposition (JI), jejunal pouch interposition (JPI) and double-tract reconstruction (DTR) (7). Kamikawa and tube-like stomach were also considered as promising methods. However, we compared only three of these reconstruction methods in our study.

## Data Availability

The original contributions presented in the study are included in the article/Supplementary Material, further inquiries can be directed to the corresponding author/s.
